# Are Food Animals Responsible for Transfer of Antimicrobial-Resistant *Escherichia coli* or Their Resistance Determinants to Human Populations? A Systematic Review

**DOI:** 10.1089/fpd.2017.2411

**Published:** 2018-08-01

**Authors:** Dishon Muloi, Melissa J. Ward, Amy B. Pedersen, Eric M. Fèvre, Mark E.J. Woolhouse, Bram A.D. van Bunnik

**Affiliations:** ^1^ Usher Institute of Population Health Sciences & Informatics, University of Edinburgh, Edinburgh, United Kingdom.; ^2^ Centre for Immunity, Infection and Evolution, School of Biological Sciences, University of Edinburgh, Edinburgh, United Kingdom.; ^3^ Nuffield Department of Clinical Medicine, University of Oxford, John Radcliffe Hospital, Oxford, United Kingdom.; ^4^ Institute of Infection and Global Health, University of Liverpool, Liverpool, United Kingdom.; ^5^ International Livestock Research Institute, Nairobi, Kenya.

**Keywords:** antimicrobial resistance, *Escherichia coli*, food animals, humans, systematic review

## Abstract

The role of farm animals in the emergence and dissemination of both AMR bacteria and their resistance determinants to humans is poorly understood and controversial. Here, we systematically reviewed the current evidence that food animals are responsible for transfer of AMR to humans. We searched PubMed, Web of Science, and EMBASE for literature published between 1940 and 2016. Our results show that eight studies (18%) suggested evidence of transmission of AMR from food animals to humans, 25 studies (56%) suggested transmission between animals and humans with no direction specified and 12 studies (26%) did not support transmission. Quality of evidence was variable among the included studies; one study (2%) used high resolution typing tools, 36 (80%) used intermediate resolution typing tools, six (13%) relied on low resolution typing tools, and two (5%) based conclusions on co-occurrence of resistance. While some studies suggested to provide evidence that transmission of AMR from food animals to humans may occur, robust conclusions on the directionality of transmission cannot be drawn due to limitations in study methodologies. Our findings highlight the need to combine high resolution genomic data analysis with systematically collected epidemiological evidence to reconstruct patterns of AMR transmission between food animals and humans.

## Introduction

The evolution of microbial pathogens that enables them to evade antimicrobial treatment has been regarded as a serious public health threat (Davies, 2011; WHO, 2015; O'Neill, 2016).

At present, the role of farm animals in the emergence and dissemination of both antimicrobial resistance (AMR) bacteria and their resistance determinants to humans is poorly understood and controversial (Marshall and Levy, 2011; Woolhouse *et al.*, 2015). Various studies have suggested that AMR bacteria and their AMR determinants can be transmitted from food animals to humans via direct contact and/or through animal products (Howells and Joynson, 1975; Aminov and Mackie, 2007; Jakobsen *et al.*, 2010; Overdevest *et al.*, 2011; Kluytmans *et al.*, 2013; Voets *et al.*, 2013). However, most of these studies have relied heavily on traditional microbiology and molecular tools, such as pulsed-field gel electrophoresis (PFGE) and multilocus sequence typing (MLST). These tools may not have sufficient discriminatory power to provide evidence of the transmission (or not) of resistant bacteria and their AMR determinants and, importantly, to infer the direction of the transmission (de Been *et al.*, 2014; Woolhouse *et al.*, 2015). Two key pathways of transfer of resistant bacteria and their AMR determinants from food animals to humans have been hypothesized: (i) horizontal transmission of AMR genes of food animal origin and (ii) clonal transfer of resistant bacteria of food animal origin to humans (Lipsitch *et al.*, 2002; Chang *et al.*, 2015). Evidence from a recent systematic review suggests that a proportion of human cephalosporin-resistant *Escherichia coli (E. coli)* clones, often associated with human disease, originate from food animals through food products (Lazarus *et al.*, 2015), though these products could have been contaminated elsewhere in the production chain (Wooldridge, 2012).

Evidence either supporting or refuting the claim that dissemination of AMR bacteria or their resistance determinants from food animals to humans is occurring will be key to the development of effective policies on antibiotic stewardship and infection control for both human and animal health. To address this knowledge gap, we performed a systematic review to (i) explore the current evidence that food animals are of the source of resistant *E. coli* and their AMR determinants in humans, (ii) examine and summarize the kinds of evidence used to support, or not support, transfer of resistant *E. coli* and their AMR determinants to humans, and (iii) make recommendations for future studies to address this question. *E. coli* is found in both human and food animal populations (Neidhardt *et al.*, 1996), and it has recently been categorized as one of the priority pathogens that pose the greatest threat to human health due to widespread AMR (WHO, 2017). It is for these reasons that, when considering transmission between hosts, we chose to focus on *E. coli*.

## Methods

### Data sources and search strategy

A systematic literature search according to the PRISMA (Preferred Reporting Items for Systematic reviews and Meta-Analyses) guidelines (Liberati *et al.*, 2009) was performed. Searches were carried out in multiple electronic databases: PubMed, Web of Science, and EMBASE for research articles published between 1940 and 2016; and Scopus for research articles published between 1960 and 2016 without geographical and language restriction. We did initial and subsequent keyword searches with various combinations of search terms: *E. coli*, AMR terminologies, human, and food animal descriptors (Supplementary Data; Supplementary Data are available online at www.liebertpub.com/fpd).

### Selection criteria and data extraction

Articles were included if they comprised an original research published in a peer reviewed journal, and investigated transmission of resistant *E. coli* and/or AMR determinants between humans and food animals. Articles were excluded if (i) they reported only agents other than *E. coli*; (ii) they studied nonfood animals; (iii) they focused exclusively on food animals or humans without any overlap between the two populations and/or (iv) they focused exclusively on food of animal origin. Article searches and screening were performed by considering article titles and abstracts for inclusion according to the search criteria. Data extraction from studies was performed by one author (D.M.M.) and independently checked by another author (B.v.B.) using a customized checklist.

### Data analysis

For all included studies we categorized the direction of AMR transmission according to the authors' conclusions: (i) studies suggesting to provide evidence of transmission from food animals to humans with direction specified; (ii) studies suggesting to provide evidence of transmission from humans to food animals with direction specified; (iii) studies suggesting overlap indicating the possibility of between-host AMR transmission, with no direction specified; and (iv) studies suggesting no evidence of transmission in either direction.

The quality of evidence was assessed using a customized Grading of Recommendations Assessment, Development and Evaluation (GRADE) system (Godfray *et al.*, 2013). Each article was matched to the following categories: (i) high resolution typing: studies using whole genome sequencing (WGS) and phylogenetic analysis; (ii) intermediate resolution typing: studies carrying out genetic characterisation through molecular tools such as MLST; (iii) low resolution typing: studies using tools such as PFGE; or (iv) co-occurrence of resistances: studies comparing AMR phenotypes between the two populations.

Additionally, we assessed the methodological quality of the articles included in the review by adapting a standardized quality assessment (Centre for Reviews Dissemination, 2009). Each article was evaluated based on two items aimed at assessing potential biases including study design (a ctive, passive) and spatiotemporal matching (no matching, temporal matching only, spatial matching only, and both temporal and spatial matching).

Because of heterogeneity of the studies (regarding typing tools, antibiotics investigated and quality of evidence) we did not perform a meta-analysis. However, we used Fisher's exact tests using R package “stats” (R Core Team, 2017) to describe associations between direction of transmission, selection bias variables and nature of transmission (clonal, determinant or both). We considered *p* < 0.05 to be statistically significant.

## Results

### Description of included studies

**FIG. 1.**
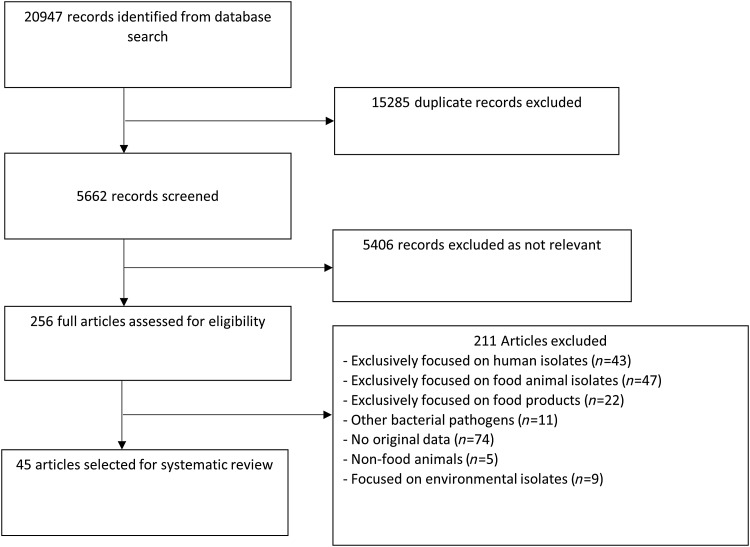
Flow diagram showing the selection of studies for inclusion.

**FIG. 2.**
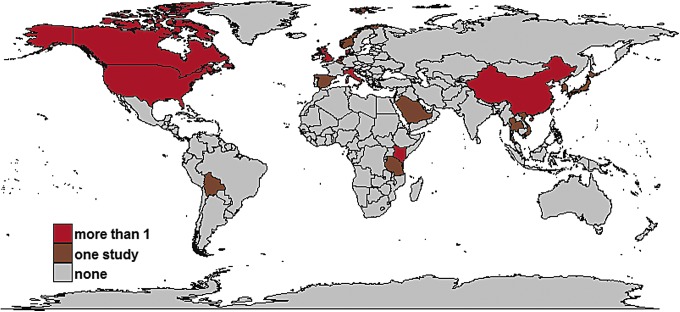
Geographic distribution of included studies. Different colors show the number of articles from each country. The map was created using several R packages [ggplot2 (Wickham *et al.*, 2013), mapdata (Becker and Wilks, 2016), maps (Becker and Wilks, 2017), and ggmap (Kahle and Wickham, 2013)] in R version 3.4.1. The shapefile with borders of countries is freely available from the Natural Earth data set (www.naturalearthdata.com). Color images available online at www.liebertpub.com/fpd

Of the 5662 distinct articles retrieved, 256 studies were reviewed (Fig. 1); and 45 studies met all inclusion criteria (Supplementary Table S1 in the Supplementary Data). The 45 studies were geographically diverse and included 20 countries, with 26 from Europe, 11 from Asia, five from North America, two from Africa, and one from the Middle East (Fig. 2).

Although the studies span five decades, there has been an increasing number of studies on this subject in recent years; with 56% of the studies published since 2010 (Supplementary Fig. S1 in the Supplementary Data). Twenty two studies (49%) had both temporal and spatial matching for human and food animal sampling, while seven (16%) had temporal matching only, and 16 (35%) were not temporally or spatially matched. We found no statistical associations between whether direction of transmission was inferred and study design or spatiotemporal matching.

Studies in our review reported different livestock species, either alone or in combination with other species. Of the eight studies that suggested transfer of AMR from food animals to humans, seven studies were based on poultry isolates and one study on pig isolates (Supplementary Fig. S2 in the Supplementary Data). Among the studies, 13 antibiotic classes were reported, either alone or in combination with other classes (Supplementary Fig. S3 in the Supplementary Data).

**FIG. 3.**
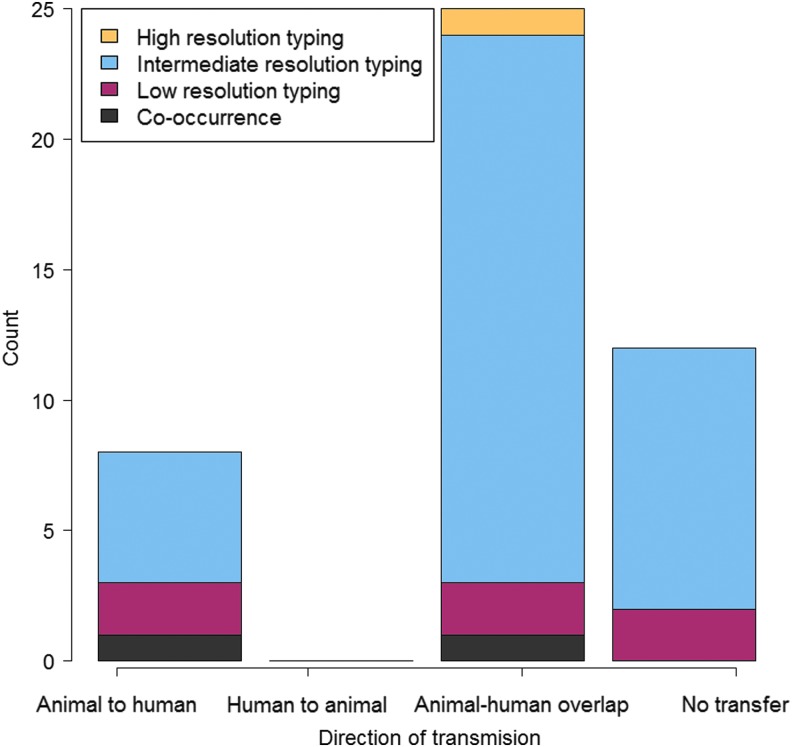
Nature of evidence used to infer direction of transmission in each study. Color images available online at www.liebertpub.com/fpd

Overall, eight studies (18%) suggested to have data to support transfer of AMR bacteria and/or their AMR determinants from food animals to humans (Levy, 1978; Al-Ghamdi *et al.*, 1999; van den Bogaard *et al.*, 2001; Hammerum *et al.*, 2006; Johnson *et al.*, 2006; Leverstein-van Hall *et al.*, 2011; Giufre *et al.*, 2012; Dierikx *et al.*, 2013), while 25 studies (56%) presented data showing overlap of AMR bacteria and AMR determinants between food animals and humans, indicating the possibility of between-host AMR transmission but with no direction specified (Jorgensen, 1983; Oppegaard *et al.*, 2001; Winokur *et al.*, 2001; Ho *et al.*, 2009, 2010; Moodley and Guardabassi, 2009; Mulvey *et al.*, 2009; Smet *et al.*, 2009; Zhang *et al.*, 2009; Jakobsen *et al.*, 2010, 2011; Zhao *et al.*, 2010; Deng *et al.*, 2011; Vieira *et al.*, 2011; Stokes *et al.*, 2012; Ciccozzi *et al.*, 2013; Hu *et al.*, 2013; de Been *et al.*, 2014; Hammerum *et al.*, 2014; Valentin *et al.*, 2014; Dahms *et al.*, 2015; Dohmen *et al.*, 2015; Huijbers *et al.*, 2015; Lupindu *et al.*, 2015; Tseng *et al.*, 2015), and 12 studies (26%) did not suggest to find evidence supporting transmission between food animals and humans (Kariuki *et al.*, 1997, 1999; Maynard *et al.*, 2004; Kang *et al.*, 2005; Phongpaichit *et al.*, 2007; Graziani *et al.*, 2009; Schwaiger *et al.*, 2010; Xia *et al.*, 2010; Johnson *et al.*, 2012; Riccobono *et al.*, 2012; Jakobsen *et al.*, 2015; Ueda *et al.*, 2015). No study in our review suggested to provide evidence for AMR transmission from humans to food animals (Fig. 3).

Only one study (2%) based its conclusion regarding transmission on high resolution typing tools, 36 studies (80%) on intermediate resolution typing tools, six (13%) on low resolution typing tools, and two (5%) on co-occurrence of resistances (Fig. 3). Overall, 18 (40%) studies based their conclusion on transmission of AMR determinants, nine (20%) on transmission of AMR bacteria, and 18 (40%) transmission of AMR bacteria together with AMR determinants (Supplementary Fig. S4 in the Supplementary Data). We found no statistical association between whether direction of transmission was inferred and the nature of transmission (*p* = 0.33).

### Studies suggesting to provide evidence of transmission of AMR from food animals to humans with direction specified

Three studies suggested to find evidence for transfer of AMR bacteria from food animals to humans, two of which concluded there is transfer of resistant clones from poultry to humans (Al-Ghamdi *et al.*, 1999; van den Bogaard *et al.*, 2001). In addition to overlapping clonal patterns, one study reported that human and chicken isolates were resistant to spectinomycin, an antibiotic mostly used in veterinary medicine (Al-Ghamdi *et al.*, 1999). Similarly, one study (van den Bogaard *et al.*, 2001) reported a higher prevalence of ciprofloxacin resistance among food animal isolates compared to human isolates.

One study found identical ciprofloxacin-resistant isolates in chicken and humans, which they concluded was suggestive of food animal to human AMR transmission (Johnson *et al.*, 2006). Two studies suggested to find evidence for horizontal transfer of AMR determinants from food animals to humans (Hammerum *et al.*, 2006; Dierikx *et al.*, 2013). One study found that clonally unrelated poultry and human isolates shared ESBL/AmpC genes located on identical plasmid families (Dierikx *et al.*, 2013). Another study found that sulfonamide-resistant isolates from pigs and healthy humans shared sul1 and sul2 genes (Hammerum *et al.*, 2006).

Three studies suggested to support transmission of both AMR bacteria and their AMR determinants from food animals to humans. Two studies found similar sequence types, plasmid families and ESBL genes in *E. coli* isolates sourced from poultry and human patients (Leverstein-van Hall *et al.*, 2011; Giufre *et al.*, 2012). A further study reported an increase in tetracycline-resistant *E. coli* in humans in contact with tetracycline fed chicken and, therefore, suggested that chicken were a reservoir of AMR bacteria and plasmids for humans (Levy, 1978).

We found that studies suggesting to provide evidence of transmission of AMR from food animals to humans did not have distinct features compared to those suggesting overlap of resistance, with regard to study methodologies, food animal species, typing tools, or antibiotics tested. For most of these it is unclear why they suggested evidence of directional transmission when 25 broadly similar studies suggested only overlap of resistance.

### Studies suggesting overlap indicating the possibility of between-host AMR transmission, with no direction specified

Four studies suggested there was evidence of overlap of resistant *E. coli* between humans and food animals. One of these studies found human and avian sequence types associated with multidrug resistance clustered together in a Bayesian phylogenetic tree (Ciccozzi *et al.*, 2013). Another study found indistinguishable PFGE patterns of ampicillin and tetracycline-resistant isolates in cattle and humans (Lupindu *et al.*, 2015). A cluster analysis of *E. coli* phylogroups found that human, pig, and chicken isolates clustered together (Jakobsen *et al.*, 2010). One extensive ecological study reported a significant correlation between the prevalence of resistance in human and livestock isolates, for both cephalosporins and fluoroquinolones (Vieira *et al.*, 2011).

Thirteen studies suggested there was evidence of overlap of AMR determinants in human and food animal isolates. Of the 13 studies, one study based on WGS and plasmid reconstruction found that clonally unrelated human and poultry isolates carried ESBL genes encoded on genetically identical plasmids (de Been *et al.*, 2014). Eleven studies found that unrelated human and food animal isolates shared identical AMR genes, integrons and plasmids (Oppegaard *et al.*, 2001; Winokur *et al.*, 2001; Ho *et al.*, 2009, 2010; Moodley and Guardabassi, 2009; Mulvey *et al.*, 2009; Smet *et al.*, 2009; Zhang *et al.*, 2009; Stokes *et al.*, 2012; Huijbers *et al.*, 2015; Tseng *et al.*, 2015). One study identified identical plasmids encoding chloramphenicol resistance in unrelated human and food animal isolates (Jorgensen, 1983).

Eight studies suggested there was evidence of overlap of resistant *E. coli* and AMR determinants, with five of these finding that clonally related human and food animal isolates harbored similar ESBL gene types and plasmid types (Hu *et al.*, 2013; Hammerum *et al.*, 2014; Valentin *et al.*, 2014; Dahms *et al.*, 2015; Dohmen *et al.*, 2015). Likewise, two studies found that clonally related human and food animal isolates carried similar fluoroquinolone AMR genes (Zhao *et al.*, 2010; Deng *et al.*, 2011). In one study, cluster analysis of AMR gene profiles and *E. coli* pathotypes showed that human and food animal isolates clustered together (Jakobsen *et al.*, 2011).

### Studies suggesting no evidence of transmission of AMR between humans and food animals

Two studies found no evidence for transfer of resistant clones, with one of these studies finding that human and avian ciprofloxacin-resistant *E. coli* strains had distinct phylogenetic compositions (Graziani *et al.*, 2009). Likewise, a PFGE analysis of multidrug-resistant *E. coli* isolates from sympatric children and chicken found that the isolates were source specific (Kariuki *et al.*, 1999).

Three studies reported no evidence for transfer of AMR determinants between food animals and humans with one of these studies reporting that human and porcine isolates had different distribution patterns of sulfonamide and tetracycline resistance genes (Schwaiger *et al.*, 2010). Two studies (Kariuki *et al.*, 1997; Phongpaichit *et al.*, 2007) reported that human and food animal multidrug-resistant isolates had distinct plasmids and integrons.

Seven studies reported no evidence for transmission of bacterial clones together with AMR determinants between food animals and humans. These studies showed that human and food animal isolates belonged to different phylogenetic groups, and had different AMR genes and plasmid profiles (Maynard *et al.*, 2004; Kang *et al.*, 2005; Xia *et al.*, 2010; Johnson *et al.*, 2012; Riccobono *et al.*, 2012; Jakobsen *et al.*, 2015; Ueda *et al.*, 2015).

## Discussion

We performed a systematic review to explore the evidence that food animals are responsible for the transfer of AMR *E. coli* and their AMR determinants to humans. Some studies in our review suggested to provide evidence for the transfer of AMR from and between food animals and humans, while a larger number did not suggest to provide evidence of transmission in either direction. In addition to the differing nature of methods used to infer direction, studies in our review differed in sampling methodologies and antibiotics tested. These differences may have affected the conclusions made regarding the epidemiological connection between food animals and humans.

Much of the evidence regarding transfer of AMR was based on the demonstration that AMR *E. coli* clones and AMR determinants were indistinguishable in both food animal and human isolates. However, the demonstration of overlapping patterns should be interpreted with care as the direction of transmission is difficult to infer, and co-colonization from a shared source is also possible. Demonstrating the direction of transmission and thus the epidemiological history of pathogens and their determinants requires a quantitative description of relatedness, including phylogenetic analysis (Grad and Lipsitch, 2014).

Molecular techniques, such as MLST and PCR, used in most studies in our review, are limited in resolution (Didelot *et al.*, 2014). In one study, *E. coli* isolates were considered genetically indistinguishable based on MLST suggesting clonal transfer (Leverstein-van Hall *et al.*, 2011); however, subsequent WGS revealed that the isolates were genetically distinct (de Been *et al.*, 2014), highlighting the need for sequencing the entire genome, rather than only a few loci. WGS provides the current “gold standard” resolution for studying genetic relatedness, but as it is a technology that has only recently become routinely available it was used in just one study in our review. Future studies in this area could benefit from combining phylogeographic methods with WGS, which yields the potential for quantitative hypothesis testing for inferring pathogen movement between host populations (De Maio *et al.*, 2015; Woolhouse *et al.*, 2015).

Just over half of the studies in our review did not consider spatiotemporal relationships between human and food animal isolates, a fundamental requirement for investigating transmission (Singer *et al.*, 2006). Future research on the directionality of transmission will benefit from designing studies in which epidemiologically linked human and food animal populations are systematically sampled, preferably longitudinally (Woolhouse *et al.*, 2015). Moreover, there is considerable diversity within both human populations (i.e., healthy individuals vs. hospitalized patients) and food animals (i.e., free range vs. intensive farming) and the specific population considered may impact their exposure to diverse groups of bacteria; thus we recommend that future studies investigating transmission of AMR between humans and food animals clearly clarify the subpopulations studied. In addition, inclusion of detailed data on antibiotic usage in these populations should be considered.

None of the included studies provided a detailed overview of antibiotic usage in either human or food animal populations, or association between antibiotic usage and subsequent development of AMR. A recent systematic review has indicated that interventions that limit antibiotic use in food animals are associated with a reduction of AMR development in humans (Tang *et al.*, 2017), and therefore further research is warranted to explore this complex association.

Although transfer of AMR from humans to food animals is likely (Barber, 2001; Wooldridge, 2012), none of the studies in our review suggested to find evidence to support transmission from humans to animals. In many instances, responsibility for the burden of AMR has been placed on food animals (Barber, 2001; Woolhouse *et al.*, 2015; Mendelson *et al.*, 2017), and thus study bias may exist in terms of source attribution. Therefore, more research is needed to provide evidence for this potential route of transfer and, importantly, the relative magnitude of that spread.

Akin to the studies in our review, most AMR studies focus on a single bacterial type; however, rapid dissemination of AMR determinants frequently occurs between bacterial species, making it hard to track infection source (Sheppard *et al.*, 2016). Tracking these determinants, frequently located on plasmids, using traditional molecular techniques may be limited. Using long read sequencing technologies such as Pacbio can overcome this by accurately generating plasmid structures (Orlek *et al.*, 2017).

Our systematic review excluded studies focusing on transmission of resistant bacteria and/or their AMR determinants through food animal-sourced food products. However, we acknowledge the potentially significant role played by food products of food animal origin in dissemination of AMR as reported in a recent systematic review (Lazarus *et al.*, 2015).

We have highlighted studies that suggest to provide evidence for transfer of resistant *E. coli* and their AMR determinants from food animals to humans. However, differences in study methodologies, such as lack of spatiotemporal overlap in sample collection, and the quality of typing tools used, suggest that transmission may occur, the evidence used to support the hypothesis is rarely compelling. The underlying problem is that demonstrating similarity or identity of AMR bacteria and/or AMR resistance determinants does not, by itself, provide information on directionality of transfer; this could be in either direction, or both, or neither but from a different source. Information on differential prevalence of resistance, and consumption of antibiotics, in the two populations may make stronger inference possible, but these data are rarely available.

Taken together, by combining genomic data analysis and epidemiological approaches it may be possible to reconstruct the complex transmission dynamics of resistant bacteria and their AMR determinants between human and food animal populations. Although we still have some way to go before a truly comprehensive integration of data—differential antibiotic usage data, detailed denominator data, information about the origin of the samples, human-food animal contact data, and pathogen sequence data—is available, disentangling and quantifying transmission of resistant bacteria and their AMR determinants between humans and food animals may still be an attainable goal.

## Supplementary Material

Supplemental data
