# Supplementary material for: Are Food Animals Responsible for Transfer of Antimicrobial-Resistant Escherichia coli or Their Resistance Determinants to Human Populations? A Systematic Review
Source: Foodborne Pathog Dis. 2018 Aug 1;15(8):467–74. doi: 10.1089/fpd.2017.2411 (PMC6103250; doi:10.1089/fpd.2017.2411)
Supplement: Supplemental data [file Supp_Data.pdf]

Supplementary Data

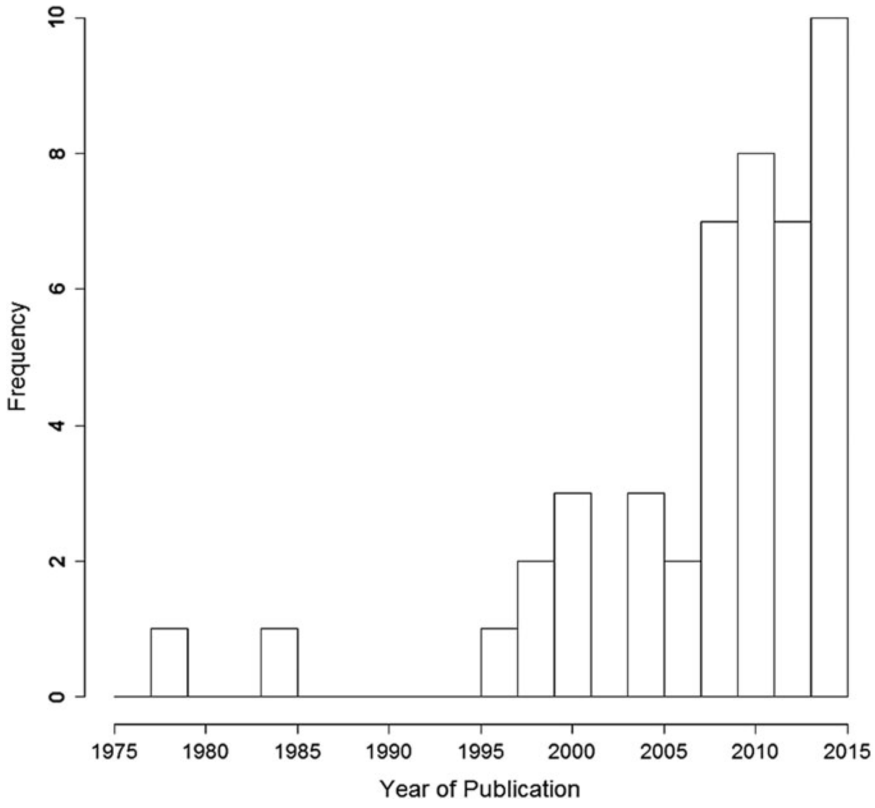

SUPPLEMENTARY FIG. S1. Annual frequency of studies in our review

Data Search Strategies

Search strategy for PubMed

(((((((((((((antibiotic resistan*) OR antimicrobial resistan*) OR drug resistan*) AND *Escherichia coli*) OR *E. coli*) OR Enterobacteria*) AND human*) AND livestock) OR food animal*) OR pig*) OR swine) OR poultry) OR bovine*) OR cattle) OR cow*)

Search strategy for Web of Science and SCOPUS

(antibiotic resistan*) OR (antimicrobial resistan*) OR (drug resistan*) AND (*Escherichia coli*) OR (*E. coli*) OR (Enterobacteria*) AND (human*) AND (livestock) OR (food animal*) OR (pig*) OR (swine) OR (poultry) OR (bovine) OR (cattle) OR (cow*)

Search strategy for EMBASE

1. antibiotic resistance/exp
2. antibiotic resistance/exp
3. drug resistance/exp
4. *Escherichia coli*/exp
5. Enterobacteria/exp
6. human/exp
7. livestock/exp
8. food animal/exp
9. pig/exp
10. swine/exp
11. poultry/exp
12. bovine/exp
13. cattle/exp
14. cow/exp

15 1 or 2 or 3

17. 4 or 5
18. 7 or 8 or 9 or 10 or 11 or 12 or 13 or 14
19. 6 and 15 and 17 and 18

References

Al-Ghamdi MS, El-Morsy F, *et al.* Antibiotic resistance of *Escherichia coli* isolated from poultry workers, patients and chicken in the eastern province of Saudi Arabia. Trop Med Int Health 1999;4:278–283.

Ciccozzi M, Giufre M, *et al.* Phylogenetic analysis of multidrug-resistant *Escherichia coli* clones isolated from humans and poultry. New Microbiol 2013;36:385–394.

Dahms C, Hubner NO, *et al.* Occurrence of ESBL-Producing *Escherichia coli* in Livestock and Farm Workers in Mecklenburg-Western Pomerania, Germany. PLoS One 2015;10:e0143326.

de Been M, Lanza VF, *et al.* Dissemination of Cephalosporin Resistance Genes between *Escherichia coli* Strains from Farm Animals and Humans by Specific Plasmid Lineages. PLoS Genet 2014;10:e1004776.

Deng Y, Zeng Z, *et al.* Dissemination of IncFII plasmids carrying rmtB and qepA in *Escherichia coli* from pigs, farm

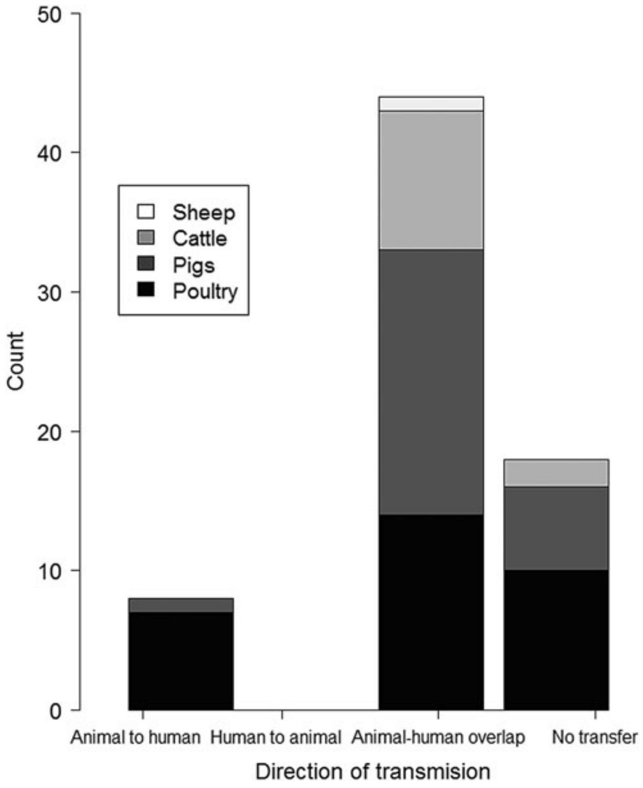

SUPPLEMENTARY FIG. S2. Counts of food animal species reported.

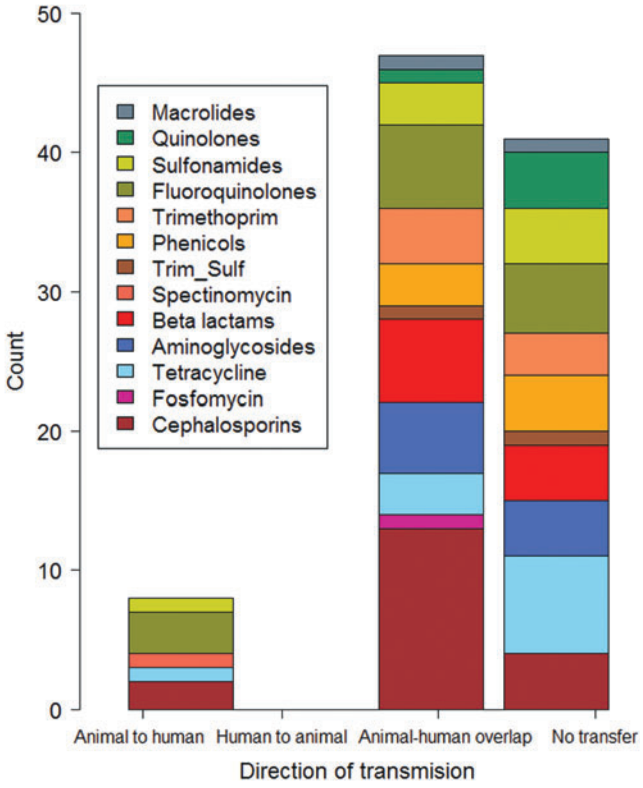

SUPPLEMENTARY FIG. S3. Counts of antibiotics reported. Note that some studies reported more than one antibiotic. (*Trim_Sulf = Trimethoprim/Sulfamethoxazole)

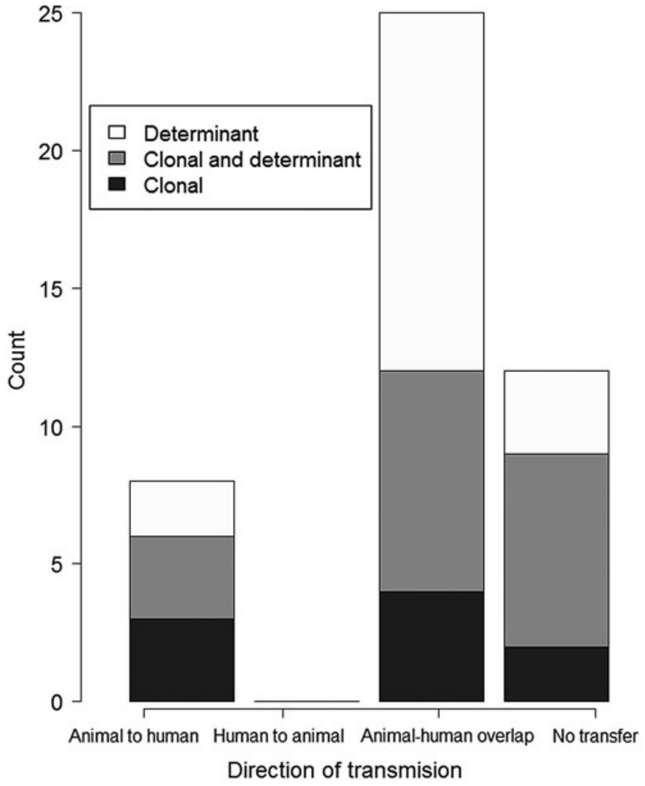

SUPPLEMENTARY FIG. S4. Nature of transfer.

workers and the environment. Clin Microbiol Infect 2011;17:1740–1745.

Dohmen W, Bonten MJM, *et al.* Carriage of extended-spectrum beta-lactamases in pig farmers is associated with occurrence in pigs. Clin Microbiol Infect 2015;21:917–923.

Giufre M, Graziani C, *et al.* *Escherichia coli* of human and avian origin: Detection of clonal groups associated with fluoroquinolone and multidrug resistance in Italy. J Antimicrob Chemother 2012;67:860–867.

Hammerum AM, Larsen J, *et al.* Characterization of extended-spectrum [beta]-lactamase (ESBL)-producing *Escherichia coli* obtained from Danish pigs, pig farmers and their families from farms with high or no consumption of third- or fourth-generation cephalosporins. J Antimicrob Chemother 2014;69:2650–2657.

Hammerum AM, Sandvag D, *et al.* Detection of sul1, sul2 and sul3 in sulphonamide resistant *Escherichia coli* isolates obtained from healthy humans, pork and pigs in Denmark. Int J Food Microbiol 2006;106:235–237

Ho PL, Wong RC, *et al.* Distribution of integron-associated trimethoprim-sulfamethoxazole resistance determinants among *Escherichia coli* from humans and food-producing animals. Lett Appl Microbiol 2009;49:627–634.

Ho P-L, Wong RC, *et al.* Genetic identity of aminoglycoside-resistance genes in *Escherichia coli* isolates from human and animal sources. J Med Microbiol 2010;59:702–707.

Hu YY, Cai JC, *et al.* Molecular typing of CTX-M-Producing *Escherichia coli* isolates from environmental water, swine feces, specimens from healthy humans, and human patients. Appl Environ Microbiol 2013;79:5988–5996.

Huijbers PMC, van Hoek AHAM, *et al.* Methicillin-resistant Staphylococcus aureus and extended-spectrum and AmpC beta-lactamase-producing *Escherichia coli* in broilers and in people living and/or working on organic broiler farms. Vet Microbiol 2015;176:120–125.

Jakobsen L, Bortolaia V, *et al.* Limited similarity between plasmids encoding CTX-M-1 beta-lactamase in *Escherichia coli* from humans, pigs, cattle, organic poultry layers and horses in Denmark. J Global Antimicrob Resist 2015;3:132–136.

Jakobsen L, Garneau P, *et al.* Microarray-based detection of extended virulence and antimicrobial resistance gene profiles in phylogroup B2 *Escherichia coli* of human, meat and animal origin. J Med Microbiol 2011;60:1502–1511.

Jakobsen L, Kurbasic A, *et al.* *Escherichia coli* isolates from broiler chicken meat, broiler chickens, pork, and pigs share phylogroups and antimicrobial resistance with community-dwelling humans and patients with urinary tract infection. Foodborne Pathogens Dis 2010;7:537–547.

Johnson JR, Kuskowski MA, *et al.* Similarity between human and chicken *Escherichia coli* isolates in relation to ciprofloxacin resistance status. J Infect Dis 2006;194:71–78.

Johnson TJ, Logue CM, *et al.* Associations between multidrug resistance, plasmid content, and virulence potential among extraintestinal pathogenic and commensal *Escherichia coli* from humans and poultry. Foodborne Pathogens Dis 2012;9:37–46.

Jorgensen ST. Relatedness of chloramphenicol resistance plasmids in epidemiologically unrelated strains of pathogenic *Escherichia coli* from man and animals. J Med Microbiol 1983;16:165–173.

Kariuki S, Gilks CF, *et al.* Plasmid diversity of multi-drug-resistant *Escherichia coli* isolated from children with diarrhoea in a poultry-farming area in Kenya. Ann Trop Med Parasitol 1997;91:87–94.

Leverstein-van Hall MA, Dierikx CM, *et al.* Dutch patients, retail chicken meat and poultry share the same ESBL genes, plasmids and strains. Clin Microbiol Infect 2011;17:873–880.

Levy SB. Emergence of antibiotic-resistant bacteria in the intestinal flora of farm inhabitants. J Infect Dis 1978;137:689–690.

Maynard C, Bekal S, *et al.* Heterogeneity among virulence and antimicrobial resistance gene profiles of extraintestinal *Escherichia coli* isolates of animal and human origin. J Clin Microbiol 2004;42:5444–5452.

Oppegaard H, Steinum TM, *et al.* Horizontal transfer of a multidrug resistance plasmid between coliform bacteria of human and bovine origin in a farm environment. Appl Environ Microbiol 2001;67:3732–3734.

Riccobono E, Pallecchi L, *et al.* Carriage of antibiotic-resistant *Escherichia coli* among healthy children and home-raised chickens: A household study in a resource-limited setting. Microb Drug Resist 2012;18:83–87.

Smet A, Martel A, *et al.* Comparative analysis of extended-spectrum-β-lactamase-carrying plasmids from different members of Enterobacteriaceae isolated from poultry, pigs and humans: Evidence for a shared β-lactam resistance gene pool? J Antimicrob Chemother 2009;63:1286–1288.

Stokes MO, Cottell JL, *et al.* Detection and characterization of pCT-like plasmid vectors for bla(CTX-M-14) in *Escherichia coli* isolates from humans, turkeys and cattle in England and Wales. J Antimicrob Chemother 2012;67:1639–1644.

Tseng S-P, Wang S-F, *et al.* Characterization of Fosfomycin Resistant Extended-Spectrum beta-Lactamase-Producing *Escherichia coli* Isolates from Human and Pig in Taiwan. PLoS One 2015;10:e0135864.

Valentin L, Sharp H, *et al.* Subgrouping of ESBL-producing *Escherichia coli* from animal and human sources: An approach to quantify the distribution of ESBL types between different reservoirs. Int J Med Microbiol 2014;304:805–816.

van den Bogaard AE, London N, *et al.* Antibiotic resistance of faecal *Escherichia coli* in poultry, poultry farmers and poultry slaughterers. J Antimicrob Chemother 2001;47:763–771.

Vieira AR, Collignon P, *et al.* Association Between Antimicrobial Resistance in *Escherichia coli* Isolates from Food Animals and Blood Stream Isolates from Humans in Europe: An Ecological Study. Foodborne Pathogens Dis 2011;8:1295–1301.

Winokur PL, Vonstein DL, *et al.* Evidence for transfer of CMY-2 AmpC beta-lactamase plasmids between *Escherichia coli* and Salmonella isolates from food animals and humans. Antimicrob Agents Chemother 2001;45:2716–2722.

Xia L-N, Li L, *et al.* A Survey of Plasmid-Mediated Fluoroquinolone Resistance Genes from *Escherichia coli* Isolates and Their Dissemination in Shandong, China. Foodborne Pathogens Dis 2010;7:207–215.

Zhang XY, Ding LJ, *et al.* Occurrence and characteristics of class 1 and class 2 integrons in resistant *Escherichia coli* isolates from animals and farm workers in northeastern China. Microb Drug Resist 2009;15:323–328.

Zhao J, Chen Z, *et al.* Prevalence and Dissemination of oqxAB in *Escherichia coli* Isolates from Animals, Farmworkers, and the Environment. Antimicrob Agents Chemother 2010;54:4219–4224.

Supplementary Table S1. Summary of 45 Included Studies

Author, Year of publication	*Sample origin and collection date*	*Source and No. of animal isolates*	*Source and No. of human isolates*	*Matching*	*Study type*	*Antibiotics reported*	*Methods used*	*Direction stated*	*Nature of evidence*	*Nature of transfer*	*Evidence for nature of transfer*
Al-Ghamdi *et al.*, 1999	Saudi Arabia, 1999	Poultry, 119	Clinical, 100<br>Healthy, 117	No matching	Active	Beta lactams, Chloramphenicol, Gentamicin, Spectinomycin, Tetracycline, Nitrofurantoin, Trimethoprim/ sulfamethoxazole, Ceftazidime	AST and Serotyping	Animal to human	Low resolution typing	Clonal	Overlapping serotypes<br>High spectinomycin resistance in humans and poultry isolates despite limited use in humans
Ueda, 2015	Vietnam, 2013	Poultry, 47	Healthy, 199	Spatial and temporal	Active	Cephalosporins	AST, PCR for gene detection, MLST for genotypic relatedness and replicon typing for plasmid characterization	No transfer	Intermediate resolution typing	Clonal and determinant	Distinct clonal, ARGs, and plasmid profiles
Jakobsen *et al.* 2015	Denmark, 2006–2010	Cattle, 4<br>Poultry, 3<br>Pigs, 21<br>Horses,15	Clinical, 22	Spatial and temporal	Active	Cephalosporins	PFGE for genotypic relatedness and PCR-based replicon typing and pMLST and restriction fragment length polymorphism (RFLP) for plasmid characterization	No transfer	Intermediate resolution typing	Clonal and determinant	Distinct clonal and plasmid profiles
Tseng, *et al.* 2015	Taiwan, 2013	Pigs, 22	Clinical, 123	Spatial and temporal	Active	Cephalosporins, Fosfomycin	AST, PFGE, and MLST for genotypic isolate relatedness, PCR for gene characterization and plasmid characterization	Animal-human overlap	Intermediate resolution typing	Determinant	Distinct clonal profiles
Lupindu, 2015	Tanzania, 2012	Cattle, 50<br>Soil, 21<br>Water, 7	Healthy, 40	Spatial and temporal	Active	Beta lactams, Tetracycline	AST and PFGE for isolate genotypic relatedness	Animal-human overlap	Low resolution typing	Clonal	Overlapping clonal profiles
Huijbers *et al.*, 2015	Netherlands, 2011–2012	Poultry, 70	Healthy, 27	Spatial and temporal	Active	Cephalosporins	AST, MLST for isolate genotypic typing and PCR for gene characterization	Animal-human overlap	Intermediate resolution typing	Determinant	Overlapping ARG profiles
Valentin *et al.*, 2014	Germany, 2011–2014	Cattle, 120<br>Poultry, 49<br>Pigs, 139	Clinical, 422<br>Healthy, 213	No matching	Active	Cephalosporins	AST, PCR for gene and isolate genotypic characterization	Animal-human overlap	Intermediate resolution typing	Clonal and determinant	Overlapping clonal and ARG profiles
Hammerum *et al.*, 2014	Denmark, 2010–2011	Pigs, 339	Healthy, 195	Spatial and temporal	Active	Cephalosporins	AST, PCR for gene detection, PFGE and MLST for isolate genotypic typing, and PBRT for plasmid replicon typing	Animal-human overlap	Intermediate resolution typing	Clonal and determinant	Overlapping clonal and ARG profiles
Hu *et al.*, 2013	China, 2012–2013 & archived isolates	Pigs, 31<br>Water, 26	Clinical, 36<br>Healthy, 46	No matching	Active & Passive	Cephalosporins	AST, PCR for gene detection, PFGE, and MLST for isolate genotypic typing	Animal-human overlap	Intermediate resolution typing	Clonal and determinant	Overlapping clonal and ARG profiles
Ciccozzi *et al.*, 2013	Italy, 2009–2010	Poultry, 43	Clinical, 129	No matching	Passive	Beta lactams, Trimethoprim/ sulfamethoxazole, Gentamicin, Cephalosporins Ciprofloxacin	AST, phylogenetic analysis of MLST sequence type sequences	Animal-human overlap	Intermediate resolution typing	Clonal	Overlapping clonal profiles
Dierikx, 2013	Netherlands, 2009	Poultry, 26	Healthy, 18	Temporal	Active	Cephalosporins	AST, Microarray analysis and PCR for gene characterization, MLST for isolate genotypic typing and RFLP for plasmid typing	Animal to human	Intermediate resolution typing	Determinant	High prevalence of resistance in poultry isolates compared to human isolates<br>Overlapping ARG and plasmid profiles

(*continued*)

Author, Year of publication	*Sample origin and collection date*	*Source and No. of animal isolates*	*Source and No. of human isolates*	*Matching*	*Study type*	*Antibiotics reported*	*Methods used*	*Direction stated*	*Nature of evidence*	*Nature of transfer*	*Evidence for nature of transfer*
Stokes, *et al.* 2012	United Kingdom, 2006–2010	Cattle, 33<br>Poultry, 9<br>Sheep, 2	Clinical, 26	No matching	Passive	Cephalosporins	PFGE and DNA arrays for isolate typing, and RFLP for plasmid characterization	Animal-human overlap	Intermediate resolution typing	Determinant	Overlapping plasmid profiles
Johnson, *et al.* 2012	USA, Netherlands and Japan, 1990–2005	Broilers and Poultry, 1331	Patients, 629<br>Healthy, 252	No matching	Passive	Sulfonamides, Tetracyclines, Macrolides, Beta lactams, Chloramphenicol, Ciprofloxacin, Gentamicin, Quinolones,	AST, PCR for ARGs and virulence genes characterization, and PRBT for plasmid typing	No transfer	Intermediate resolution typing	Clonal and determinant	Distinct clonal, ARG and, plasmid profiles
Deng *et al.*, 2011	China, 2002	Pigs, 24<br>Environment, 5	Healthy, 12	Spatial and temporal	Passive	Fluoroquinolones	AST, PCR for ARGs characterization, PFGE and MLST for isolate genotypic typing, and PBRT for plasmid replicon typing	Animal-human overlap	Intermediate resolution typing	Clonal and determinant	Overlapping clonal, ARG, and plasmid profiles
Zhao *et al.*, 2010	China, 2002	Poultry, 25<br>Pigs, 73	Healthy, 33	Spatial and temporal	Active	Quinolones, Ciprofloxacin	AST, PCR for ARGs characterization, PFGE and MLST for isolate genotypic typing, and RLFP for plasmid typing	Animal-human overlap	Intermediate resolution typing	Clonal and determinant	Overlapping clonal and ARG profiles
Schwaiger, 2010	Germany, 2002–2004	Pigs, 137	Patients,152	No matching	Active	Tetracyclines, Sulfonamides, Streptomycin	AST, PCR for ARGs characterization	No transfer	Intermediate resolution typing	Determinant	Distinct ARG profiles
Moodley, 2009	Denmark, 2007	Pigs, 70<br>Environment, 35	Healthy, 5	Spatial and temporal	Active	Cephalosporins	AST, PCR for ARGs characterization, PFGE for isolate genotypic typing and, RLFP for plasmid typing	Animal-human overlap	Intermediate resolution typing	Determinant	Overlapping plasmid profiles
Mulvey, 2009	Canada, 1999–2000	Cattle,51	Patients, 25	Temporal	Passive	Cephalosporins	AST, PCR for ARGs characterization, PFGE for isolate genotypic typing and plasmid typing	Animal-human overlap	Intermediate resolution typing	Determinant	Overlapping plasmid profiles
Ho *et al.*, 2009	China, 2002–2004	Cattle, 11<br>Poultry, 25<br>Pigs, 28	Patients, 51<br>Healthy, 59	No matching	Passive	Trimethoprim-Sulfamethoxazole	AST, PCR for ARGs and integron characterization, and PFGE for isolate genotypic typing	Animal-human overlap	Intermediate resolution typing	Determinant	Overlapping integron and ARG profiles
Graziani, 2009	Italy, 2006	Poultry, 113	Healthy, 125	No matching	Passive	Ciprofloxacin	AST, PCR, and PFGE for isolate genotypic and virulence gene typing	No transfer	Intermediate resolution typing	Clonal	Distinct clonal profiles
Hammerum *et al.*, 2006	Denmark, 2002–2003	Pigs, 610<br>Pork, 189	Healthy, 199	No matching	Active	Sulfonamides	AST, PCR for ARGs characterization	Animal to human	Intermediate resolution typing	Determinant	Overlapping ARGs in animal and human isolates<br>High prevalence of resistance in animal isolates compared to human isolates
Johnson *et al.*, 2006	Spain, 1996–1998	Poultry, 49	Healthy, 68	Spatial and temporal	Passive	Ciprofloxacin	AST, PCR, and PFGE for genotypic and virulence gene typing	Animal to human	Intermediate resolution typing	Clonal	High similarity in resistant animal and human isolates
Maynard *et al.*, 2004	Canada, 2001	Pigs, poultry, cattle and pets, 39	Patients, 70	No matching	Active	Beta lactams, Aminoglycosides, Tetracycline, Phenicols, Trimethoprim, Sulfonamides	AST,PCR, and colony hybridization for ARGs characterization and distribution	No transfer	Intermediate resolution typing	Clonal and determinant	Distinct clonal profiles

(*continued*)

Supplementary Table S1. (Continued)

Author, Year of publication	*Sample origin and collection date*	*Source and No. of animal isolates*	*Source and No. of human isolates*	*Matching*	*Study type*	*Antibiotics reported*	*Methods used*	*Direction stated*	*Nature of evidence*	*Nature of transfer*	*Evidence for nature of transfer*
Kang, 2005	South Korea, 2001–2003	Poultry, 163<br>Pigs, 133	Patients,201<br>Healthy,167	Temporal	Active	Trimethoprim, Aminoglycosides, Sulfonamides	AST, bPCR for integron characterization, PFGE for genotypic typing, and plasmid typing	No transfer	Intermediate resolution typing	Clonal and determinant	Distinct clonal and plasmid profiles
Oppegaard *et al.*, 2001	Norway, 1996	Cattle, 13	Healthy, 7	Spatial and temporal	Active	Beta lactams, Tetracycline, Sulfonamides, Streptomycin, Trimethoprim	AST, PCR for integron characterization, serotyping, and plasmid typing	Animal-human overlap	Intermediate resolution typing	Determinant	Overlapping plasmid profiles
van den Bogaard *et al.*, 2001	Netherlands, 2001	Poultry, 122	Healthy, 216	Spatial and temporal	Active	Ciprofloxacin	AST, PFGE for isolate genotypic typing	Animal to human	Low resolution typing	Clonal	High prevalence of resistance in animal isolates compared to human isolates<br>Overlapping clonal profiles
Kariuki, 1999	Kenya, 1994	Poultry, 248	Healthy,128	Spatial and temporal	Active	Beta lactams, Aminoglycosides, Tetracycline, Chloramphenicol, Trimethoprim, Ciprofloxacin, Quinolones,	AST, PFGE for isolate genotypic typing, and plasmid typing	No transfer	Low resolution typing	Clonal	Distinct clonal profiles
Leverstein-van Hall *et al.*, 2011	Netherlands, 2006–2010	Poultry, 35<br>Poultry meat, 81	Patients,409	No matching	Passive	Cephalosporins	AST, microarray analysis for ARGs characterization, MLST for isolate genotypic typing, and PBRT for plasmid typing	Animal to human	Intermediate resolution typing	Clonal and determinant	Overlapping clonal, ARG, and plasmid profiles
Jakobsen *et al.*, 2011	Denmark, 2004	Poultry, 17<br>Pigs, 8<br>Pork, 30<br>Broiler meat,18	Patients, 52<br>Healthy, 36	Spatial and temporal	Active	Beta lactams, Chloramphenicol, Trimethoprim, Ciprofloxacin, Quinolones, Macrolides	Microarray analysis for ARGs and virulence genes characterization	Animal-human overlap	Intermediate resolution typing	Clonal and determinant	Overlapping clonal and ARG profiles
Vieira *et al.*, 2011	Europe, 2005–2008	Poultry, 4000<br>Pigs, 4500<br>Cattle, 3500	Patients, 100,000	Temporal	Passive	Beta lactams, Ciprofloxacin, Aminoglycosides, Cephalosporins	AST	Animal-human overlap	Co-occurrence	Clonal	Significant correlations between human and animal isolates
Ho *et al.*, 2010	China, 2002–2004	Cattle, 11<br>Poultry, 37<br>Pigs, 33<br>Fish, 1	Patients, 167	No matching	Active	Aminoglycosides	AST, PCR for ARGs characterization, PCR and PFGE for isolate genotypic typing, and PBRT for plasmid typing	Animal-human overlap	Intermediate resolution typing	Determinant	Overlapping ARG and plasmid profiles
Xia *et al.*, 2010	China, 2007	Poultry,198<br>Pigs, 137	Patients,139<br>Healthy,18	Spatial and temporal	Active	Fluoroquinolones	AST, PCR for ARGs characterization, PCR and PFGE for isolate genotypic typing	No transfer	Intermediate resolution typing	Clonal and determinant	Distinct clonal patterns
Dohmen *et al.*, 2015	Netherlands, 2011	Pigs, 160	Healthy, 13	Spatial and temporal	Active	Cephalosporins	AST, PCR for ARGs characterization, PCR and MLST for isolate genotypic typing, and PBRT for plasmid typing	Animal-human overlap	Intermediate resolution typing	Clonal and determinant	Overlapping clonal, ARG, and plasmid profiles
Kariuki, *et al.* 1997	Kenya, 1993–1994	Poultry, 246	Patients,168	Spatial and temporal	Active	Beta lactams, Tetracycline, Chloramphenicol, Cephalosporins, Ciprofloxacin, Quinolones,	AST and restriction endonuclease digestion (RED) for plasmid typing	No transfer	Low resolution typing	Determinant	Distinct clonal patterns

(*continued*)

Supplementary Table S1. (Continued)

Author, Year of publication	*Sample origin and collection date*	*Source and No. of animal isolates*	*Source and No. of human isolates*	*Matching*	*Study type*	*Antibiotics reported*	*Methods used*	*Direction stated*	*Nature of evidence*	*Nature of transfer*	*Evidence for nature of transfer*
Phongpaichit, 2007	Thailand, 2004–2005	Pigs, 432	Healthy, 185	Spatial and temporal	Active	Beta lactams, Aminoglycosides, Tetracycline, Chloramphenicol, Cephalosporins, Trimethoprim-Sulfamethoxazole, Ciprofloxacin, Quinolones, Macrolides	AST and PCR for ARGs and integron characterization	No transfer	Intermediate resolution typing	Determinant	Distinct integron patterns
Riccobono*et al.*, 2012	Bolivia, 2002–2005	Poultry, 36	Healthy, 30	Spatial and temporal	Active	Tetracyclines, Quinolones	AST and PCR for ARGs characterization and genotypic typing	No transfer	Intermediate resolution typing	Clonal and determinant	Distinct clonal and plasmid profiles
Zhang *et al.*, 2009	China, 2004–2007	Poultry, 106<br>Pigs, 172	Healthy, 23	Spatial and temporal	Active	Aminoglycosides, Trimethoprim, Sulfonamides	AST and PCR for ARGs and integron characterization	Animal-human overlap	Intermediate resolution typing	Determinant	Overlapping ARG and integron profiles
Levy, 1978	USA 1974	Poultry, 320	Healthy, 337	Spatial and temporal	Active	Tetracyclines	AST	Animal to human	Low resolution typing	Clonal and determinant	Overlapping clonal profiles
de Been *et al.*, 2014	Netherlands, 2006–2011	Poultry, 4<br>Poultry meat, 7<br>Pig, 4	Healthy, patients, 17	No matching	Passive	Cephalosporins	AST, MLST, and WGS for genotypic typing, and phylogenetics for relatedness analysis	Animal - human overlap	High resolution typing	Determinant	Increase in antibiotic-resistance in humans in contact with both tetracycline-fed chicken and oxytetracycline supplemented feed
Jorgensen, 1983	Denmark, Britain 1960–1981	Pigs, 19	Patients, 13	No matching	Passive	Cephalosporins	AST and hybridization techniques for plasmid analysis	Animal-human overlap	Low resolution typing	Clonal and determinant	Overlapping plasmid profiles
Dahms *et al.*, 2015	Germany, 2012	Cattle, 10<br>Pig, 33<br>Poultry, 13	Healthy, 5	Spatial and temporal	Active	Beta lactam, Chloramphenicol, Ciprofloxacin, Streptomycin, Sulfamethoxazole, Tetracycline, Trimethoprim	AST, PCR for ARGs characterization, MLST for isolate genotypic typing	Animal-human overlap	Intermediate resolution typing	Clonal	Overlapping clonal and ARG profiles
Jakobsen *et al.*, 2010	Denmark, 2004–2006	Broiler meat, 283<br>Pork, 187<br>Poultry, 138<br>Pigs, 145	Patients, 102<br>Healthy, 109	Spatial and temporal	Active	Ciprofloxacin	AST, PCR for genotypic typing	Animal to human	Intermediate resolution typing	Clonal and determinant	Overlapping clonal profiles
Giufre *et al.*, 2012	Italy, 2009	Poultry, 101	Patients, 277	Temporal	Passive	Chloramphenicol	AST, PCR for ARGs characterization, PCR and MLST for isolate genotypic typing	Animal-human overlap	Intermediate resolution typing	Determinant	Overlapping clonal profiles
Winokur *et al.*, 2001	USA, 1998–2000	Cattle &pigs, 377	Patients, 1017	Temporal	Active	Beta lactams	AST, isoelectric focus analysis for ARGs characterization, PFGE for genotypic typing and RFLP for plasmid analysis	Animal-human overlap	Intermediate resolution typing	Determinant	Overlapping plasmid and ARG profiles
Smet *et al.*, 2009	Belgium, 2001–2007	Poultry, 4<br>Pigs, 2	Healthy, 3	No matching	Active	Cephalosporins	AST, PCR, and isoelectric focus analysis for ARGs characterization, and PBRT and RFLP for plasmid analysis	Animal-human overlap	Intermediate resolution typing	Determinant	Overlapping plasmid and ARG profiles

AST, antibiotic susceptibility testing; PCR, polymerase chain reaction; ARGs, antibiotic resistance genes; MLST, multilocus sequence typing; PFGE, pulsed-field gel electrophoresis; RFLP, restricted fragment length polymorphism; pMLST plasmid multilocus sequence typing; PBRT, PCR-based replicon typing.
